# Optical Coherence Tomography: An Adjunctive Tool for Differentiating between Choroidal Melanoma and Metastasis

**DOI:** 10.1155/2016/9803547

**Published:** 2016-02-22

**Authors:** Vicktoria Vishnevskia-Dai, Dinah Zur, Shiran Yaacobi, Iris Moroz, Hadas Newman, Meira Neudorfer

**Affiliations:** ^1^Ocular Oncology and Autoimmune Center, The Goldschleger Eye Institute, Sheba Medical Center, 52621 Ramat Gan, Israel; ^2^Division of Ophthalmology, Tel Aviv Medical Center, 64239 Tel Aviv, Israel

## Abstract

*Purpose.* To investigate the value of optical coherence tomography (OCT) for differentiation between choroidal melanoma and metastasis based on characteristics of the anterior choroidal surface and the chorioretinal interface.* Methods.* This retrospective observational case series included 29 patients with untreated choroidal melanomas and 21 patients with untreated choroidal metastases. Regularity and lobularity characteristics of the anterior choroidal surface were evaluated in a masked manner. Retinal and retinal pigment epithelium (RPE) findings were documented as well.* Results.* OCT demonstrated a regular and smooth anterior choroidal surface in 89.7% of the eyes with melanoma and in 47.6% of the eyes with metastasis (*p* = 0.002; sensitivity = 89.7%; specificity = 52.4%). The anterior choroidal contour was lobulated in 81.0% of the eyes with metastasis versus 17.2% of the eyes with melanoma (*p* < 0.001; sensitivity = 82.8%; specificity = 81.0%). RPE thickness and neuroretinal characteristics (e.g., retinal thickness, the presence of cysts, and the presence of subretinal fluid) were similar in both choroidal tumors.* Conclusion*. OCT may serve as a noninvasive adjunctive tool for the differential diagnosis of choroidal tumors. Choroidal melanomas usually demonstrate regular surfaces on OCT, while choroidal metastases usually have an irregular and lobulated surface.

## 1. Introduction

The diagnosis of a choroidal tumor is based mainly on clinical characteristics. Additional diagnostic testing includes ultrasonography (US), fluorescein angiography, and color Doppler testing [1]. US enables localization of the intraocular mass, estimation of its size, and characterization of its tissue texture. Choroidal melanomas are characterized by a dome-shaped mass on B-scans and by low-to-medium homogeneous internal reflectivity on A-scans. In contrast, choroidal metastases are characterized by a flatter or slightly dome-shaped mass on B-scans and by medium-to-high nonhomogeneous internal reflectivity on A-scans. They are mostly multilobular with an irregular surface [2–5]. The differentiation between the two tumor entities is often challenging, especially in cases of atypical findings or small tumors (<3 mm height), which could clinically appear similar. Although a fine-needle biopsy can provide definitive tissue diagnosis, it is widely avoided because of the risk of intraocular injury like vitreous hemorrhage, retinal tears, and others. Furthermore, biopsy is limited by the necessity of a qualified cytologist, insufficiency or inadequacy of the retrieved material, delay in diagnosis, and higher costs [6].

Therefore, there is a need for a risk-free, credible, and readily available diagnostic tool for the differential diagnosis and assessment of intraocular lesions.

Optical coherence tomography (OCT) uses near-infrared light in order to deliver high-resolution cross-sectional images of the retina which are very similar to the histopathological specimens [7]. During the last decade, OCT became a crucial diagnostic tool in retinal practice due to its simplicity, noninvasiveness, and plenty of information.

Retinal and retinal pigment epithelium (RPE) changes overlying choroidal melanomas and metastases have been comprehensively described by time-domain and spectral-domain optical coherence tomography (SD-OCT) [2, 8–10].

Both entities show similar changes, which can include neurosensory retinal detachment, retinal cystoid degeneration, highly reflective lesions overlying the RPE-choriocapillaris complex, intraretinal splitting and abnormal retinal structure, RPE thickening, and irregularity of the RPE. Since the changes of the overlying retina and RPE are not pathognomonic to the underlying disease, they do not assist in the differential diagnosis between metastasis and melanoma. Enhanced depth imaging OCT (EDI-OCT) has been recently used to evaluate choroidal metastasis [11, 12] and melanoma [13–15]. The purpose of this study was to investigate whether OCT features of the anterior contour and the chorioretinal interface overlying choroidal melanoma and choroidal metastasis may assist in the differentiation between the tumor types.

## 2. Methods

The records of the ophthalmology departments in two large medical centers in Israel, the Tel-Aviv Sourasky Medical Center and the Sheba Medical Center, were retrospectively reviewed with the approval of the hospitals' review boards. Patients diagnosed as having either choroidal melanoma or choroidal metastasis from 1/1/2007 to 1/31/2014 were reviewed. Only patients with a technically decipherable OCT scan displaying the anterior surface of the tumor and the overlying retina were included in the study. Exams acquired with time-domain OCT were included only in cases where image quality allowed evaluation of all imaging parameters defined below.

OCT of the tumors of all sizes were included in the study only if they depicted the anterior surface of the tumor and overlying retina. No limitation for tumor size was placed since we did not aim to image the posterior portion of the tumor. However, tumors that were too high to display the anterior surface and overlying retina were excluded. Patients whose tumors had been treated previously were excluded from the study, as were patients with other posterior segment disease or surgery or treatment.

The tumor type diagnosis was made by an ocular oncologist and was based on patient medical history, clinical examination, and imaging findings. The data was retrieved from the medical files. The recorded parameters included demographic information, clinical presentation, US findings, and oncologic data. Choroidal tumors were evaluated for clinical features including quadrantic location, maximal basal diameter and height, pigmentation, overlying RPE alterations, subretinal fluid, lipofuscin pigment, and drusen. Echographic features of the tumors included thickness, configuration (plateau/dome/mushroom), echogenicity (solid/hollow), internal reflectivity (high/medium/low), and subretinal fluid (absent or present).

Patients with other posterior segment disease or surgery or treatment were excluded.

OCT was performed using distinct technologies by the Spectralis system (Heidelberg Engineering, Heidelberg, Germany), the Cirrus HD-OCT unit (Carl Zeiss Meditec, Dublin, CA), and the Stratus system (Zeiss Meditec, Dublin, CA). The patient's baseline OCT at presentation was chosen for a sequential qualitative analysis. After a review of all scans acquired, the scan that most optimally demonstrated the anterior tumor surface was chosen for evaluation. The anterior choroidal surface at the site of the tumor was classified according to its regularity and lobularity. The regularity of the tumor surface was assessed subjectively based on the experience of the authors. The overlying retina was investigated for cystoid or noncystoid retinal edema, subretinal fluid, retinal thinning, and the status of the photoreceptors. The evaluation of the imaging parameters was done by a single reader who is an experienced ophthalmologist specializing in ophthalmic imaging. The reading ophthalmologist was masked to the diagnosis of the tumor type. Major outcome measures were anterior surface regularity and lobularity. Minor outcome measures were changes of the retina and RPE overlying the choroidal tumor.

### 2.1. Statistical Analysis

Data were described as averages with standard deviations for continuous variables and by frequencies with percentages for categorical variables. The association between dichotomous variables and diagnosis was tested by Fisher's exact test, and that between continuous variables and diagnosis was tested by the* t*-test. All calculations were performed using SPSS statistical software.

## 3. Results

Fifty eyes of 50 consecutive patients were included in the study. 29 had a malignant choroidal melanoma and 21 had choroidal metastasis. The patients' demographic features are listed in Table 1. There were 5 cases of bilateral metastasis, but only one eye of each patient was included in the study since the location of the tumor in the other eye did not allow visualization with OCT. One patient had two metastases in one eye; only one could be visualized by OCT and was included.

**Figure 1:**
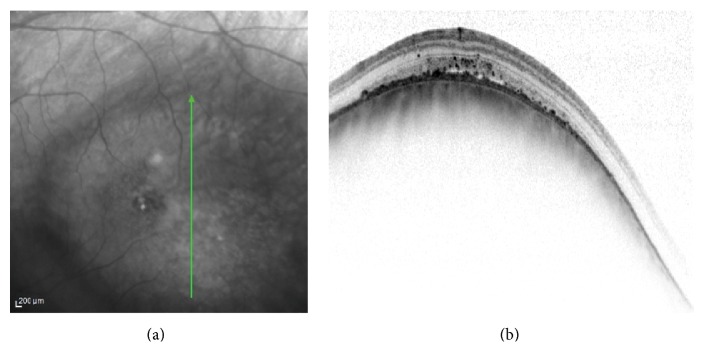
Left eye choroidal melanoma. The melanoma is demonstrated temporal to the macula in the infrared image (left). The vertical OCT (right) at the center of the tumor showing a smooth regular dome-shaped elevation of the choroid, irregular thickening of the RPE, limited subretinal fluid, and intraretinal hyperreflective spots.

**Figure 2:**
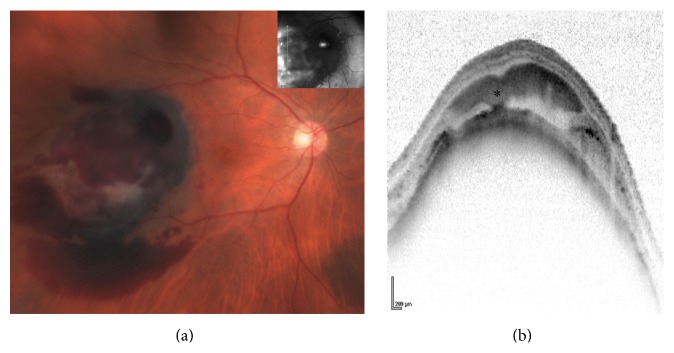
Right eye choroidal melanoma. The melanoma is demonstrated temporal to the macula with intra- and subretinal hemorrhage (a). Vertical OCT scan (b) at the temporal aspect of the tumor showing a smooth regular dome-shaped elevation of the choroid with subretinal hemorrhage (asterisk). There is hyperreflective thickening of the RPE causing shadowing; the RPE is not continuous on the inferior aspect of the tumor. Still, the choroidal elevation is smooth and regular.

**Figure 3:**
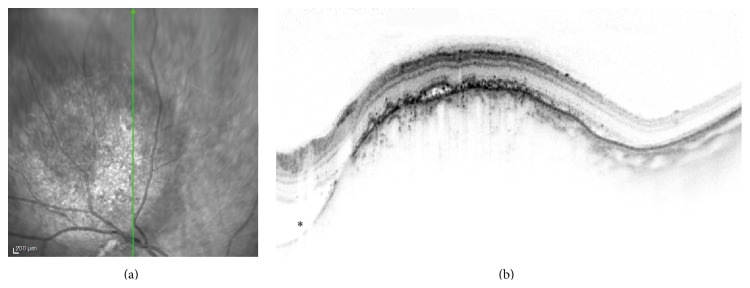
Right eye choroidal metastasis in a patient with parotid carcinoma. The amelanotic tumor is demonstrated superotemporal to the optic disc in the infrared image (a). Vertical OCT scan (b) at the center of the tumor showing an irregular anterior choroidal surface, structural loss of the inner segment ellipsoid band, and a small quantity of subretinal fluid with hyperreflective speckles. Note also the subretinal fluid adjacent to the tumor (asterisk).

**Figure 4:**
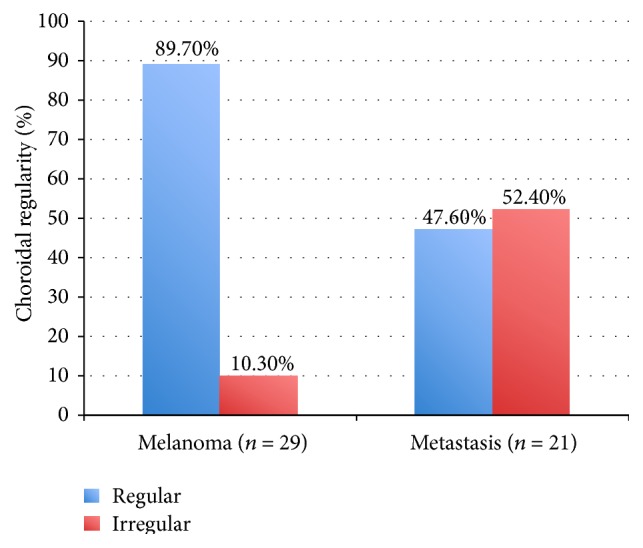
Choroidal regularity. Almost 90% of the malignant choroidal melanomas had a regular choroidal surface compared with about one-half of the choroidal metastases.

**Figure 5:**
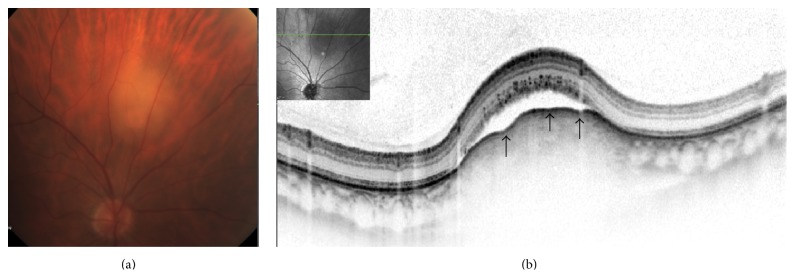
Right eye choroidal metastasis in a patient with esophageal carcinoma. The amelanotic choroidal metastasis is demonstrated superior to the optic disc (a). Horizontal OCT scan (b) at the center of the tumor showing lobularity of the anterior choroidal surface (arrows). There is subretinal fluid and structural loss of the internal/outer segment junction with shaggy photoreceptors.

**Figure 6:**
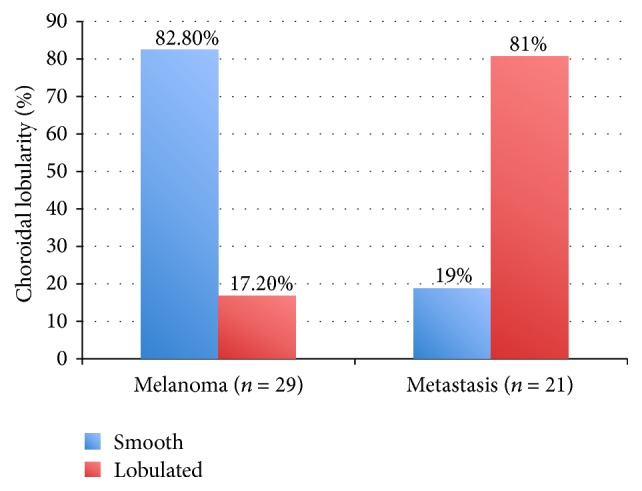
Choroidal lobularity. More than 80% of the choroidal metastases had a lobulated choroidal surface, whereas only 17% of the malignant choroidal melanomas were lobulated.

In all cases of choroidal metastasis, the primary tumor was known. The oncologic features of the choroidal metastasis are given in detail in Table 2. OCT was performed using Cirrus HD-OCT in 25 patients, Spectralis System in 19 patients, and Stratus OCT in 6 patients. The anterior choroidal surface of the tumor and the overlying retina were adequately demonstrated on all systems. Qualitative analysis, as listed in Table 3, revealed that choroidal melanomas showed a smooth regular anterior choroidal surface in 26/29 cases (89.7%) and an irregular surface in only 3/29 cases (10.3%, Figures 1 and 2). In contrast, the anterior choroidal surface in choroidal metastasis was regular in 10/21 cases (47.6%) and irregular in 11/21 cases (52.4%, Figure 3). This difference was statistically significant (*p* = 0.002; sensitivity = 89.7%, specificity = 52.4%, Figure 4). Only 5/29 (17.2%) of the tumors in the melanoma group had a lobulated anterior choroidal surface. This is in contrast to 17/21 (81.0%) of the tumors which had a lobulated choroidal surface in the metastasis group (Figure 5). This difference between the two groups was statistically significant (*p* < 0.001; sensitivity = 82.8%, specificity = 81.0%, Figure 6).

As defined by OCT, overlying retinal edema was demonstrated in 14/29 (48.3%) eyes in the melanoma group and in 8/21 (38.1%) in the metastasis group (*p* = 0.569). Only one tumor in each group had cystoid changes of the overlying retina (*p* = 1.0). Twelve of the 28 (42.9%) eyes with melanoma and 6/20 (30%) of the eyes with metastasis (*p* = 0.546) showed atrophic thinning of the neurosensory retina overlying the tumor. Most of the tumors in the melanoma group (23/29, 79.3%) showed irregularity of the RPE, and 15/29 (51.7%) of them had thickening of the RPE. Most of the tumors in the metastasis group (16/20, 80.0%) had irregularity of the RPE, and 9/20 (45.0%) showed thickening of the RPE. Subretinal fluid appeared in 22/29 (75.9%) tumors in the melanoma group and in all but one of the tumors (19/20, 95.2%) in the metastasis group. Hyperreflective spots were demonstrated in the subretinal fluid in 9 eyes with melanomas and 10 eyes with metastasis.

In the metastasis group, we did not find differences in OCT characteristics in tumors from different origins.

## 4. Discussion

Our study showed that SD-OCT presents a valuable adjunctive tool for differentiating between the choroidal melanoma and metastasis. Regularity and lobularity of the anterior tumor surface showed significant differences between the two groups.

While the well-known retinal and RPE changes are similar in choroidal melanoma and metastasis, EDI-OCT was recently used to describe the different choroidal characteristics as well as the anterior surface features of melanomas, metastasis, and lymphomas [12–16]. Our study is the first to investigate the value of SD-OCT for the differentiation between tumor types. Having two relatively large groups of patients with different choroidal tumors allowed us to compare the features of choroidal melanoma surfaces to those of choroidal metastasis in a masked manner.

Our results, acquired with SD-OCT, support the findings described earlier, using EDI-OCT [12, 13, 15]. The smooth and regular appearance of the anterior choroidal surface in choroidal melanoma in our study correlates with findings by Shields et al. [13]. Small choroidal melanomas were shown to have a smooth dome-shaped tumor surface with choriocapillaris thinning and inward compression of large choroidal vessels [13]. In contrast, metastases appear to have an irregular and lobulated contour in most of the cases. Similarly, Al-Dahmash et al. observed an irregular and “lumpy bumpy” appearance of the anterior surface in 64% of 14 metastasis cases [12]. In our study, choroidal metastasis had a similar appearance on OCT, irrespective of the primary tumor.

In cases of choroidal lymphoma, Shields et al. reported that the tumor surface varies depending on the tumor thickness [16]. Thinner choroidal infiltration was associated with smooth or calm surface, whereas increased thickness was accompanied by a mini-waxy surface or rippled surface and thickness >4 mm created an undulating waxy appearance

Although EDI-OCT is widely available, spectral domain OCT is still the sole system in many institutes. We found spectral domain OCT to be a useful tool, providing similar information regarding the anterior tumor surface.

The importance of OCT in the evaluation of choroidal tumors has been discussed by several authors who focused on the overlying retinal and RPE changes using TD-OCT [2, 8, 17, 18] and SD-OCT [19–22]. Subretinal fluid is commonly associated with choroidal melanoma with an incidence of 91–100% [8, 20] and has been shown to be a risk factor for tumor growth [17, 18]. Similarly, choroidal metastases are frequently associated with subretinal fluids (67–100%) [2, 11, 12]. We found subretinal fluid in 75% of our melanoma cases and in 95% of our metastasis cases. About one-third of the melanoma cases and one-half of the metastasis cases in this study had hyperreflective spots in the subretinal fluid. Demirci et al. interpreted those findings as shed outer segment photoreceptors in persistent subretinal fluid overlying choroidal metastasis [11]. Shields et al. described “shaggy photoreceptors” referring to irregular, elongated, and presumed swollen photoreceptors from subretinal fluid [13]. In addition, Arevalo et al. found those spots in 86% of choroidal metastases and speculated they were a result of retinal compromise by cancer or inflammatory cells [2]. Singh et al. used spectral domain OCT to describe dispersed accumulation of subretinal deposits corresponding to orange pigment over small choroidal melanoma; this finding was not demonstrated using time-domain OCT [19]. Spectral domain OCT was also capable of detecting early vitreous seeding as highly reflective 20–30 micron spheroidal bodies in the vitreous [23].

The main limitation of using OCT for imaging of choroidal melanoma lies in the difficulty of imaging the overlying retina in large melanomas and the inability to image tissue deeper to the anterior choroidal surface [19]. Reflectivity of the anterior choroid in melanomas is variable even with spectral domain OCT.

Ultrasound is the most widely used imaging modality for diagnosing choroidal tumors and differentiating between tumor entities. Its value increases as tumor thickness increases, and it is indispensable for the measurement of tumor size in larger tumors. Typical cases of melanoma display a dome-shaped tumor of low-to-medium internal reflectivity, while metastases display a lobular and irregular surface with medium-to-high nonhomogeneous internal reflectivity. Small tumors and atypical cases do not necessarily have those characteristics and diagnosis may be more difficult. Our study was not designed for comparison between echographic and OCT data. We included only patients with a definitive tumor diagnosis, which was based amongst other on US findings. OCT results were not meant to question this diagnosis. Moreover, lobularity as seen on US and on OCT is not equivalent. The resolution of ultrasonography is 50–200 *μ* [24] compared to 5 *μ* for spectral domain OCT [25]. This explains why US can show the gross tumor surface but cannot display the lobular and irregular changes found in the anterior tumor surface using OCT. These features can be especially helpful in cases of small tumors for which US information is scarce.

Two major limitations in our study are its retrospective design and the qualitative rather than quantitative evaluation of the study parameters. The analysis of the OCT images was done by an experienced ophthalmologist specializing in ophthalmic imaging, yet the subjective manner of the evaluation of the choroidal surface cannot be excluded.

Clinical evaluation and ultrasound are the mainstay and the gold standard in diagnosis of choroidal tumors. Our findings highlight the value of OCT as an adjunctive diagnostic tool for differentiation between choroidal melanoma and choroidal metastasis, by providing helpful, statistically specific, and sensitive information regarding the anterior tumor surface. OCT is a readily available tool that allows noninvasive and relatively inexpensive tumor evaluation.

## Figures and Tables

**Table 1:** Demographic features of 50 patients with choroidal tumors (50 eyes).

Feature	Melanoma (*n* = 29)	Metastases (*n* = 21)
Mean age (range), year	63 (34–88)	60 (31–87)
Gender, *n* (%)		
Male	10 (35)	9 (43)
Female	19 (65)	12 (57)
Eye effected, *n* (%)		
Right	11	10 (48)
Left	17	6 (28)
Both	0	5 (24)

**Table 2:** Oncologic features of choroidal metastases in 21 eyes of 21 patients.

Feature	Metastasis (*n* = 21)
Primary tumor, *n* (%)	
Breast	7 (33.3)
NSCLC	6 (28.6)
Other, *n* (%)	8 (38.1)
Carcinoma of tonsils	1 (4.8)
Carcinoma of thyroid	2 (9.5)
Carcinoma of esophagus	1 (4.8)
Carcinoma of parotid	1 (4.8)
Carcinoma of prostate	1 (4.8)
Renal cell carcinoma	1 (4.8)
Ewing sarcoma	1 (4.8)
Extraocular metastases, *n* (%)	
Yes	14 (67)
No	7 (33)
Presenting symptom, *n* (%)	
Ocular	3 (14)
Extraocular	18 (86)

NSCLC = non-small cell lung cancer.

**Table 3:** Optical coherence tomographic features of choroidal tumors in 50 eyes of 50 patients.

Feature	Melanoma (*n* = 29)	Metastases (*n* = 21)	*p* Value
Choroid			
Irregular, *n* (%)	3 (10)	11 (52)	0.001
Lobulated, *n* (%)	5 (17)	17 (81)	0.000
RPE			
Irregular, *n* (%)	23 (79)	16 (80)	0.855
Thickened, *n* (%)	15 (52)	9 (45)	0.568
Retina			
Thickened, *n* (%)	14 (48)	8 (38)	0.569
Cystic, *n* (%)	1 (3)	1 (5)	0.839
Atrophic NS retina, *n* (%)	12 (43)	6 (30)	0.460
Subretinal			
Subretinal fluid, *n* (%)	22 (76)	20 (95)	0.117
Hyperreflective spots, *n* (%)	9 (31)	10 (48)	0.281

RPE = retinal pigment epithelium; NS = neurosensory.

## References

[1] Neudorfer M., Waisbourd M., Anteby I. (2011). Color flow mapping: a non-invasive tool for characterizing and differentiating between uveal melanomas and choroidal metastases. *Oncology Reports*.

[2] Arevalo J. F., Fernandez C. F., Garcia R. A. (2005). Optical coherence tomography characteristics of choroidal metastasis. *Ophthalmology*.

[3] Anteby I., Pe'er J. (1993). Ultrasonography—a major imaging tool in the diagnosis of intraocular tumors. *Harefuah*.

[4] Byrne S. F., Green R. L. (1992). *Ultrasound of the Eye and Orbit*.

[5] Sobottka B., Schlote T., Krumpaszky H. G., Kreissig I. (1998). Choroidal metastases and choroidal melanomas: comparison of ultrasonographic findings. *British Journal of Ophthalmology*.

[6] Cohen V. M. L., Dinakaran S., Parsons M. A., Rennie I. G. (2001). Transvitreal fine needle aspiration biopsy: the influence of intraocular lesion size on diagnostic biopsy result. *Eye*.

[7] Costa R. A., Skaf M., Melo L. A. S. (2006). Retinal assessment using optical coherence tomography. *Progress in Retinal and Eye Research*.

[8] Muscat S., Parks S., Kemp E., Keating D. (2004). Secondary retinal changes associated with choroidal naevi and melanomas documented by optical coherence tomography. *British Journal of Ophthalmology*.

[9] Shields C. L., Materin M. A., Shields J. A. (2005). Review of optical coherence tomography for intraocular tumors. *Current Opinion in Ophthalmology*.

[10] Say E. A. T., Shah S. U., Ferenczy S., Shields C. L. (2011). Optical coherence tomography of retinal and choroidal tumors. *Journal of Ophthalmology*.

[11] Demirci H., Cullen A., Sundstrom J. M. (2014). Enhanced depth imaging optical coherence tomography of choroidal metastasis. *Retina*.

[12] Al-Dahmash S. A., Shields C. L., Kaliki S., Johnson T., Shields J. A. (2014). Enhanced depth imaging optical coherence tomography of choroidal metastasis in 14 eyes. *Retina*.

[13] Shields C. L., Kaliki S., Rojanaporn D., Ferenczy S. R., Shields J. A. (2012). Enhanced depth imaging optical coherence tomography of small choroidal melanoma. *Archives of Ophthalmology*.

[14] Torres V. L. L., Brugnoni N., Kaiser P. K., Singh A. D. (2011). Optical coherence tomography enhanced depth imaging of choroidal tumors. *American Journal of Ophthalmology*.

[15] Shields C. L., Pellegrini M., Ferenczy S. R., Shields J. A. (2014). Enhanced depth imaging optical coherence tomography of intraocular tumors: from placid to seasick to rock and rolling topography—the 2013 Francesco Orzalesi Lecture. *Retina*.

[16] Shields C. L., Arepalli S., Pellegrini M., Mashayekhi A., Shields J. A. (2014). Choroidal lymphoma shows calm, rippled, or undulating topography on enhanced depth imaging optical coherence tomography in 14 eyes. *Retina*.

[17] Espinoza G., Rosenblatt B., Harbour J. W. (2004). Optical coherence tomography in the evaluation of retinal changes associated with suspicious choroidal melanocytic tumors. *American Journal of Ophthalmology*.

[18] Shields C. L., Furuta M., Thangappan A. (2009). Metastasis of uveal melanoma millimeter-by-millimeter in 8033 consecutive eyes. *Archives of Ophthalmology*.

[19] Singh A. D., Belfort R. N., Sayanagi K., Kaiser P. K. (2010). Fourier domain optical coherence tomographic and auto-fluorescence findings in indeterminate choroidal melanocytic lesions. *British Journal of Ophthalmology*.

[20] Sayanagi K., Pelayes D. E., Kaiser P. K., Singh A. D. (2011). 3D Spectral domain optical coherence tomography findings in choroidal tumors. *European Journal of Ophthalmology*.

[21] Saxena S., Jain A., Ramindar Sharma S., Meyer C. H. (2012). Three-dimensional spectral domain optical coherence tomography of retina in choroidal metastasis due to uterine endometrial carcinoma. *BMJ Case Reports*.

[22] Iuliano L., Scotti F., Gagliardi M., Bianchi I., Pierro L. (2012). SD-OCT patterns of the different stages of choroidal metastases. *Ophthalmic Surgery, Lasers & Imaging*.

[23] Shah S. U., Kaliki S., Shields C. L., Ferenczy S. R., Harmon S. A., Shields J. A. (2012). Enhanced depth imaging optical coherence tomography of choroidal nevus in 104 cases. *Ophthalmology*.

[24] Webb S. (1988). Diagnostic ultrasound. *The Physics of Medical Imaging*.

[25] Leung C. K.-S., Cheung C. Y.-L., Weinreb R. N. (2008). Comparison of macular thickness measurements between time domain and spectral domain optical coherence tomography. *Investigative Ophthalmology & Visual Science*.

